# Progressive noradrenergic degeneration and motor cortical dysfunction in Parkinson’s disease

**DOI:** 10.1038/s41401-024-01428-z

**Published:** 2024-11-26

**Authors:** Wei Zhou, Hong-Yuan Chu

**Affiliations:** 1 Department of Pharmacology and Physiology, Georgetown University Medical Center, Washington D.C., WA 20007 USA; 2 Aligning Science Across Parkinson’s (ASAP) Collaborative Research Network, Chevy Chase, MD 20852 USA

**Keywords:** locus coeruleus, norepinephrine, dopamine, parkinson’s disease, motor cortex, cognition

## Abstract

The locus coeruleus norepinephrine (LC-NE) system plays an important role in regulating brain function, and its neuronal loss has been well-documented in Parkinson’s disease (PD). The LC-NE neurodegeneration is believed to underlie various nonmotor symptoms in people with PD, including neuropsychiatric deficits, sleep disruptions, and cognitive impairments. Of particular interest, LC-NE neurons send intensive axonal projections to the motor regions of the cerebral cortex. However, how NE depletion in the motor cortex contributes to PD pathophysiology remains poorly understood. In addition, recent studies provided increasing mechanistic insights into secondary changes in the cerebral cortex as LC-NE degenerates, which might involve its interaction with dopaminergic signaling during the chronic course of the disease. In the present article, we briefly discuss clinical and preclinical studies that support the critical roles of LC-NE neurodegeneration and motor cortical dysfunction in both motor and nonmotor deficits in Parkinsonian states. We focus our discussion on the potential impact of LC-NE neurodegeneration on motor cortical function and the subsequent symptom manifestation. Last, we propose future research directions that can advance our understanding of cortical pathophysiology in PD by integrating noradrenergic degeneration.

## Introduction

Locus coeruleus (LC) is a bilateral structure situated in the upper dorsolateral pontine tegmentum. LC neurons synthesize and release norepinephrine (NE, also known as noradrenaline), which is essential for regulating brain function. LC of both hemispheres comprises about 50,000 cells in humans and around 3000 cells in rodents [1–4]. The LC-NE neurons project to broad brain regions (e.g., the cerebral cortex, the thalamus, the amygdala, the hippocampus, the cerebellum, and the medulla) and play a pivotal role in regulating normal brain function and behavioral states, including arousal, attention, cognition, and stress responses. Noradrenergic receptors (ARs) can be categorized into 3 subtypes, including α1, α2, and β receptors, which are G-protein coupled receptors (GPCRs). While α1 and β receptors are coupled with G_q_ and G_s_ proteins, respectively, α2 receptors are coupled with G_i_ protein and the subsequent signaling cascades [5]. Particularly, α2 receptors are expressed highly at noradrenergic axon terminals, where they can act as autoreceptors to regulate presynaptic NE release. Thus, NE has a higher affinity for α2 receptors than α1 and β receptors. The basic neurobiology of LC-NE neurons, receptor signaling, and their regulation of behavior have been extensively studied and well-reviewed by others [6–9].

The potential contribution of LC-NE neurodegeneration to the pathogenesis and symptomology of PD has been reported and discussed in depth [10–12]. The present article focuses on a thorough analysis of LC-NE innervation of the cerebral cortex, particularly the cortical motor regions, at molecular and cellular levels. We further discuss potential cortical circuitry adaptations as LC-NE neurons progressively degenerate. Last, we propose the potential roles of such circuit changes in the pathophysiology of motor and cognitive deficits in PD.

## Segregated LC-NE projections to the cerebral cortical subregions

The LC was thought to be a homogeneous brain region comprising neurons that express dopamine β-hydroxylase (DBH), the enzyme that converts dopamine (DA) to NE. LC-NE neurons receive convergent inputs from various brain regions and exhibit highly divergent outputs throughout the brain [1]. The connectomic feature is consistent with the broad and uniform influence of the LC-NE system on brain activity and physiological function, e.g., arousal and wakefulness [13, 14].

**Fig. 1:**
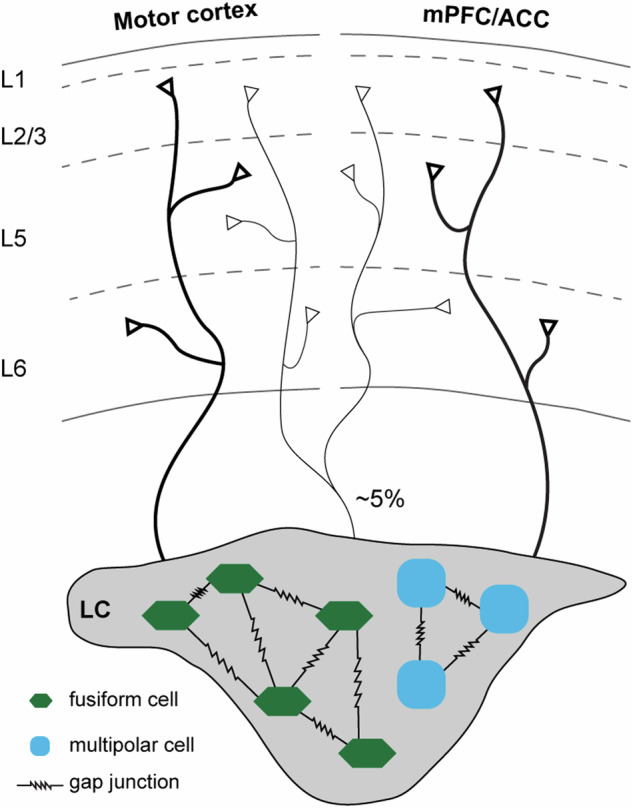
Diagram showing the heterogeneity of LC-NE neurons and their segregated outputs to cortical subregions. Modified from McKinney et al. [20] and Chandler et al. [19].

A large amount of evidence shows that LC includes a heterogeneous group of neurons with distinct molecular, cellular, and connectivity properties [6]. Using in vivo physiology recording approaches, Su et al. reported two subtypes of LC-NE neurons that can be distinguished based on the biophysical properties of their action potentials and their distinct responses to reinforcement learning [15]. It supports the ensemble encoding of the LC neurons, thereby regulating specific brain function and behavior through spatially and temporally distinct subpopulations [16]. At the microcircuit level, emerging evidence also suggests that LC-NE neurons assemble into sub-circuits and form segregated channels for targeted modulation of brain function [1, 17–19]. To dissect LC-NE neuronal subcircuits (or “ensemble”), McKinney et al. conducted sophisticated multi-patch recordings from the LC brain slices. They reported two subtypes of LC-NE neurons based on electrophysiological properties, i.e., the fusiform (~62%) and multipolar (32%) cells (Fig. 1) [2, 20]. LC-NE neurons form gap junctions within each subtype for communication, potentially segregating them into neuronal ensembles to fire together for specific functions. In line with the segregated output concept of the LC-NE system, Chandler et al. reported that only ~5% of LC-NE neurons project to both the primary motor cortex (M1) and other prefrontal subregions [e.g., the medial prefrontal cortex (mPFC) or anterior cingular cortex(ACC)] (Fig. 1) [19]. The M1-projecting LC-NE neurons exhibit distinct molecular and physiological phenotypes compared to those projecting to other cortical subregions. In conclusion, compelling evidence from molecular, physiological, and anatomical studies suggests that LC comprises a heterogeneous group of neurons that form distinct neuronal assembles and modulate their targeting brain regions through segregated outputs.

## Cellular organization of noradrenergic innervation of the cerebral cortex

The prefrontal cortex is a major target of long-range noradrenergic projections, as indicated by the high levels of DBH-immunoreactive axonal fibers in both rodents and monkeys. However, certain levels of variability exist among prefrontal subregions [21, 22]. These fibers exhibit an overall diffused distribution pattern across cortical layers, with layer 5 showing the highest level of DBH immunoreactivity [22]. At microscopic levels, only about 20% of noradrenergic varicosities (0.65 μm in diameter) form synaptic contacts with dendritic shafts or spines in the prefrontal cortex. This anatomical feature suggests that noradrenergic inputs modulate cortical function mainly through non-synaptic volume transmission. Thus, once released, NE could diffuse broadly within cortical microcircuits and regulate cortical circuit function by modulating the activity of multiple cellular targets. This conclusion is consistent with the overall observation that noradrenergic receptors can be detected in multiple circuit elements within cortical circuits.

NE receptors are broadly expressed across cortical layers II-VI of primates and rodents [23, 24]. The pattern of noradrenergic receptor expression across cortical layers matches the occupancy of cortical areas by intensive noradrenergic fibers (see above). In primates and rodents, immunofluorescent studies showed that subcortical-projecting cortical pyramidal neurons express high levels of α1, α2, β1, and β2 NE receptors [25, 26]. The expression of various subtypes of noradrenergic receptors in a cortical neuron is probably essential for NE in regulating animals’ arousal state and behavior. However, it remains unclear how distinct subtypes of noradrenergic receptors are engaged under different physiological and behavioral states.

At cellular and subcellular levels, α1 receptors are expressed in presynaptic axon terminals [27, 28], postsynaptic dendritic spines of pyramidal neurons and interneurons [27, 29, 30], and astrocytes [27] in the cerebral cortices of rodents and monkeys. In addition to being expressed at presynaptic axon terminals of noradrenergic input as autoreceptors, α2 receptors are also expressed postsynaptically on dendritic spines in layer II-III of the prefrontal cortex. These postsynaptic α2 receptors exert potent regulation of the working memory of monkeys [31]. Similarly, β1 receptors are expressed in dendritic spines of pyramidal neurons and interneurons (e.g., parvalbumin-expressing cells) in layer III of dorsolateral PFC in both NHPs and rodents [32, 33].

The literature has documented the interaction between NE and other monoamines, like DA [9]. NE and DA can be co-released at noradrenergic axon terminals in cortical regions and hippocampus with particular physiological and behavioral significance [9, 34–36]. Dopamine transporters (DAT), a protein responsible for the reuptake of synaptically released DA, are sparsely expressed in the deep layers of the prefrontal cortex [37]. Moreover, DAT is located far from the release sites at presynaptic axon terminals in the cortex. The subcellular distribution of DAT allows broader extracellular diffusion of DA (i.e., “volume transmission”) in the cerebral cortex and long-lasting stimulation of DA receptors and even noradrenergic receptors [38]. Similarly, stimulation of DA receptors by NE release from the LC area has also been documented [39]. Compelling evidence supports that NE transporters in the cortex are responsible for DA reuptake [40]. Moreover, noradrenergic α1 receptors and DA D1 receptors are colocalized in the dendrites of the prefrontal cortex [41]. Thus, NE-DA co-neurotransmission and their synergistic interactions can be critical in modulating cerebral cortical function and related behaviors.

## Noradrenergic modulation of cortical circuit activity and cognition

In the following section, we briefly discuss earlier studies on cellular mechanisms of noradrenergic regulation of the prefrontal-dependent working memory and emerging interests in noradrenergic modulation of sensorimotor integration.

### Noradrenergic regulation of working memory

Noradrenergic regulation of cognitive function was extensively studied in the prefrontal cortex-dependent working memory. Earlier in vivo electrophysiology recording studies reported that the layer III pyramidal neurons in the prefrontal cortex showed sustained firing during the delayed period in the delayed response tasks, which defined their microcircuits as the cellular basis of working memory function in primates [42–44]. NE signaling plays a critical role in modulating connections among cortical pyramidal neurons and the working memory performance, which is achieved through multiple synaptic and cellular mechanisms. Under physiological conditions, moderate levels of NE enhance the working memory by promoting persistent activities of prefrontal cortical pyramidal neurons via postsynaptic α2A receptors expressed at dendritic spines [31]. In addition, NE can further strengthen the working memory performance through the interactions of α1-α2 receptors. It has been reported that α1 receptors interact with α2 noradrenergic receptors to enhance the persistent activity of cortical pyramidal neurons by increasing presynaptic glutamate release and enhancing postsynaptic dendritic excitability [45]. Under nonphysiological states (e.g., stress), high NE levels in the cortex can impair prefrontal-dependent working memory. The detrimental effect of NE involves distinct noradrenergic receptor subtypes and molecular signaling cascades, including the β1 receptors expressed in cortical GABAergic interneurons [32, 46]. Depending on the NE levels, an inverted U-shaped dose-response relationship between cortical NE and working memory performance has been documented in the literature [7, 47]. Distinct noradrenergic receptors are linked to different intracellular signaling cascades and have different affinities to NE. Thus, levels of LC-NE neuronal activity and the amount of NE release determine the direction and magnitude of noradrenergic regulation of PFC function and cognition.

### Noradrenergic regulation of sensorimotor integration

Emerging evidence suggests that the LC-NE system plays a role in sensorimotor integration and sensory-guided motor activities [48]. The primary (M1) and secondary (M2) motor cortices in rodents receive intensive noradrenergic inputs from the LC [19, 48, 49]. While certain levels of overlap exist, LC-NE neurons projecting to M1 and PFC seem to be from distinct subtypes (Fig. 1). For example, the M1-projecting LC-NE neurons in rats exhibit slower spontaneous firing ex vivo and smaller AMPA receptor-mediated spontaneous glutamate transmission than those projecting to the PFC [19, 48]. These physiological features indicate that more convergent synaptic inputs are needed to excite M1-projecting LC-NE neurons when compared to those projecting to the PFC. The difference in the intrinsic and synaptic properties is expected to result in a smaller amount of NE release in the M1 relative to the PFC under physiological state. However, the M1 of rats showed comparable NE concentrations at the tissue level relative to the PFC [36]. Such discrepancies could be due to the behavioral states (e.g., anesthesia versus freely moving) of these animals while NE concentration was assessed. However, potential differences in other aspects of cortical noradrenergic signaling cannot be excluded (e.g., expression levels of NE transporters and autoreceptors).

At cellular and molecular levels, Schiemann et al. conducted in vivo whole-cell patch-clamp recordings and reported that LC-NE inputs maintain a tonic membrane depolarization of corticospinal neurons in layer 5 of M1 and increase their probability of firing by modulating HCN channel function [50, 51]. Behaviorally, pharmacological blockade of M1 noradrenergic receptors impaired the precision of forepaw placement in a self-paced voluntary movement of mice [51]. This study documented the critical role of noradrenergic modulation in skilled motor control, which may involve the LC-NE-induced motor cortical circuit plasticity [52]. It is worth noting that HCN channels differentially regulate the intrinsic and synaptic properties of distinct subtypes of cortical neurons, implying that LC-NE may fine-tune cortical circuit function through cell-type- and input-selective mechanisms [50, 53]. Considering the detailed analysis of noradrenergic regulation of the mPFC circuits and working memory, further studies remain to determine how NE regulates motor cortical circuit activity and contributes to the execution of motor activities.

## LC degeneration and PD symptoms

### LC neurodegeneration in post-mortem PD brains

A large amount of evidence suggests that LC-NE neurons show selective vulnerability and significant neurodegeneration (50%–80%) at early stages of PD [10, 54]. Consistently, post-mortem analysis of the brains of people with PD showed that lesions associated with the accumulation of Lewy pathology in the LC could be detected as early as the Braak stage 2 in PD, which gradually deteriorated as the disease progressed [55, 56]. In addition, it has been documented that the degeneration of LC-NE neurons is comparable to the SNc dopamine neuronal loss, confirming the concept that both the LC and SNc are heavily involved in the pathogenesis and pathophysiology of PD [54, 55, 57, 58].

**Fig. 2:**
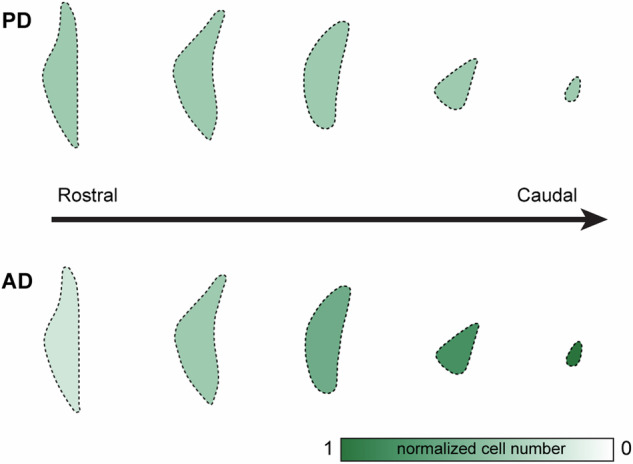
Diagram showing different patterns of LC degeneration in PD and AD. LC-NE neurons degenerate evenly throughout the rostrocaudal portions in PD, which is in contrast to the preferential degeneration of the rostral part of LC in AD. See Zarow [54] for details.

Zarow et al. conducted a semiquantitative analysis of post-mortem brains of pathologically confirmed PD cases (*n* = 19) and documented that LC showed a profound neuronal loss in PD [54]. Of particular interest is that this study reported a lack of correlation between the age of onset or the duration of disease and the number of surviving neurons in the LC in PD. Moreover, the extent of LC cell loss in PD cases showed small inter-individual variabilities, further suggesting LC neurons’ selective susceptibility in PD [54]. In contrast to the greater cell loss in the rostral portion of LC in AD, LC-NE neuronal loss in PD could be detected evenly throughout the entire structure (Fig. 2) [59]. Such disease-specific patterns of LC neurodegeneration warrant future studies to understand pathobiological processes underlying the susceptibility of LC-NE neurons between AD and PD.

The observations from post-mortem PD brains suggest that, similar to the SNc dopaminergic neurons, neuromelanin-laden LC-NE neurons are heavily involved in the development of PD and that their degeneration can be detected at an early stage of the disease progression.

The PD subtype with early LC-NE degeneration (i.e., noradrenergic PD) has been recently recognized as a distinct early disease subtype [12]. It is proposed that noradrenergic PD manifests various nonmotor deficits, including rapid eye movement sleep behavior disorder (RBD), pain, neuropsychiatric, and cognitive deficits. Extensive neuropathology and neuroimaging studies support that these nonmotor deficits are driven by LC-NE neurodegeneration and their innervation of both the central and peripheral systems (see [12] for more details). Thus, noradrenergic PD differs from PD with dominant dopaminergic degeneration in the midbrain showing classic parkinsonian motor deficits (e.g., bradykinesia and akinesia). Subtyping PD patients based on the manifestation of early phenotypes could help to design specific strategies for individuals afflicted by the disease and potentially modify the disease progression more effectively. In striking contrast to the extensive studies and cellular and circuit consequences of nigral DA degeneration to the basal ganglia network and the subsequent development of parkinsonian motor deficits, our understanding of how LC-NE degeneration affects brain function remains limited.

### LC neurodegeneration in animal models of PD

Animal model studies have documented the degeneration of LC-NE neurons and the associated detrimental consequences. Using a viral approach to overexpress human mutant A53T α-synuclein, Herich et al. reported a progressive loss of LC-NE neurons starting from 3 weeks post injections, which was accompanied by a gradual development of α-synuclein pathology in the LC [60]. In a recent study, Butkovich et al. developed transgenic mice (i.e., *DBH-hSNCA*) selectively expressing human α-synuclein in noradrenergic neurons and performed longitudinal histological and behavioral studies. Although pathologic species of α-synuclein accumulated in the LC-NE neurons, there was only a loss of NE fibers but not LC neurons at 14 months of age [61]. The transgenic mice manifested abnormal sleep patterns and increased anxiety-like behavior, which noradrenergic antagonists could rescue [61]. These observations are generally consistent with the pivotal role of LC-NE neurons in regulating the sleep-wake cycle and emotion. They are also supported by studies using animal models with LC-NE lesions [62, 63]. Thus, these mice can be a useful model to study the biological mechanisms associated with the manifestation of nonmotor deficits (e.g., anxiety and sleep disorders) as seen in prodromal PD. Loss of LC-NE neurons has also been reported in transgenic mice and rats with PD risk genes, including the PINK1 knockout and LRRK2 knockout animals [64, 65].

### LC-NE neuronal dysfunction and nonmotor deficits in PD

In parallel to the robust LC neuronal death, there was a 60%–80% reduction of NE concentration in the cerebral cortex and hippocampus in the post-mortem brains of PD patients with dementia [66]. Noradrenergic projections to the forebrain regions are essential in regulating attention, cognition, and emotion [7]. Thus, LC-NE neurodegeneration and the subsequent reduction of NE levels in cortical regions likely result in the under-stimulation of cortical noradrenergic receptors, underlying certain aspects of cognitive deficits in PD patients. In addition, coordination and synergistic interaction of DA and NE signaling in modulating cortical cellular and circuit function have been well-studied and reviewed in the mPFC [67]. For example, normal function and LC-NE projection to the cortex and its intact transmission are necessary for proper dopaminergic neurotransmission and reuptake within the cortical circuits, particularly given that the expression levels of DA transporters are low in the cortex and that DA uptake might be mediated by norepinephrine transporters [37, 40]. Thus, early LC-NE degeneration in PD is hypothesized to disrupt dopaminergic modulation of cortical function that might play a role in the manifestation of cognitive deficits and other symptoms in PD.

It is worth noting that LC-NE neurodegeneration and reduced NE concentrations in the brain trigger compensatory responses in their projecting regions. Earlier studies reported an upregulation of α1- and β1-receptors but a downregulation of α2 receptors in the frontal cortex of demented PD patients [68]. Since most α2 receptors are autoreceptors expressed presynaptically, their reduction perhaps reflects the severe loss of LC-NE axon terminals. The increased α1- and β1-receptors are supposed to be the compensation for the decreased cortical NE levels, which might play a pivotal role in developing dementia in this demented PD population. Therefore, both the reduced NE levels and dysregulated noradrenergic receptor signaling could contribute to the manifestation of cognitive impairments and emotion dysregulation in PD.

Preclinical studies using animal models may provide additional mechanistic understanding of LC-NE degeneration and the associated compensatory changes in PD. LC-NE neurons have been reported to show an accelerated firing rate in both α-synuclein-based and neurotoxin-induced models of prodromal PD. The increased firing frequency is expected to compensate for damages to the LC-NE neurons to sustain NE release and maintain NE levels at their targeting regions [62, 69]. In addition, DSP4-induced loss of NE fibers (but no loss of LC-NE neurons) has been reported to increase NE release and its turnover and increase the density and sensitivity of postsynaptic α1 and β1 adrenergic receptors [63, 70, 71]. The increased NE release was proposed to contribute to the development of anxiety-like behavior in the DSP4-treated mice [63], which is a prevalent prodromal symptom in PD. Taken together, impaired neuronal activity, dysregulated NE release, compensatory changes of receptor signaling, and the neurodegeneration of LC-NE neurons may contribute to the development of anxiety, depression, and/or cognitive deficits at distinct stages of PD.

### LC-NE dysfunction, dopaminergic neurodegeneration, and motor deficits in PD

**Fig. 3:**
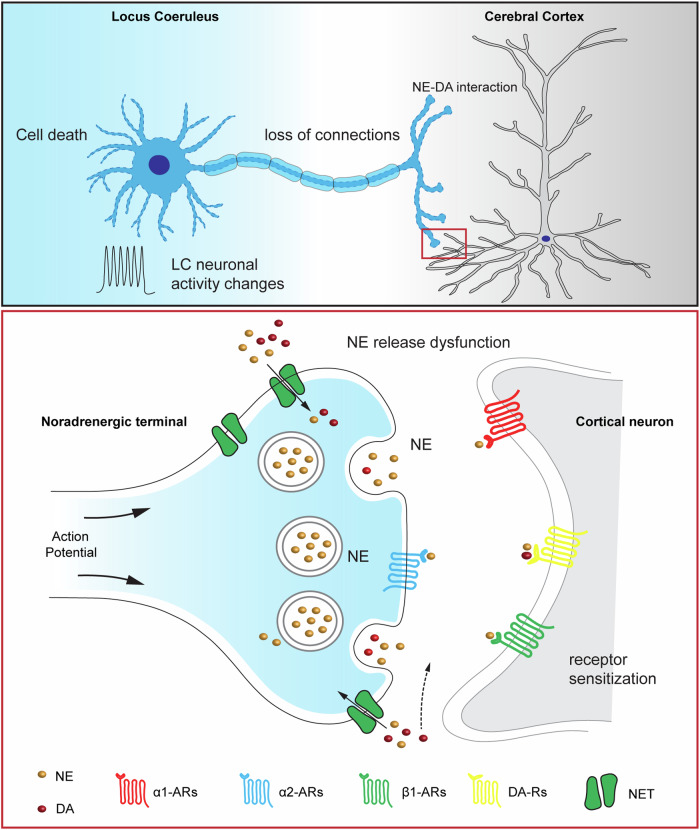
Hypothesized molecular, cellular, and circuit changes in the cerebral cortex as LC-NE neurons progressively degenerate. Before cell death in the LC region at a late stage of the disease, LC neurons likely show altered neuronal firing (i.e., compensatory), NE release alterations (i.e., compensatory), and loss of LC projections to the cortex at early stages (upper). Dysfunctional NE neurotransmission can alter local NE levels in the cortex, which may subsequently induce the sensitization of noradrenergic receptors in cortical neurons (i.e., compensatory). Importantly, altered expression levels and function of NE transporters will likely trigger secondary effects to cortical dopaminergic neurotransmission (e.g., a larger volume of DA diffusion in the cortex and prolonged DA receptor stimulation, dashed arrow) due to low dopamine transporter expression in the cortical regions.

The impact of LC-NE degeneration may also play a role in manifesting motor deficits, and the associated mechanisms are multifold (Fig. 3).

First, the LC-NE system can directly regulate motor activity through its broad projections to motor regions in the brain, including the M1 [62, 63]. LC-NE inputs are essential to maintain normal network dynamics of M1 at cellular and mesoscopic levels by stimulating cortical noradrenergic receptors [50–52]. Therefore, severe LC-NE degeneration is expected to decrease cortical NE levels significantly and impair control of fine motor activities [51]. In line with this speculation, depletion of NE by genetically selective knocking out DHB led to severe motor deficits (e.g., fine motor skills) in mice that were even more profound than that in MPTP-treated mice [72]. Second, dopaminergic modulation of cortical circuits is critical in proper motor control [73, 74]. Potential disruption of cortical dopaminergic neurotransmission associated with LC-NE degeneration (see above) likely affects cortical control of motor activity, underlying certain aspects of motor deficits in PD (Fig. 3). Third, it has been reported that LC noradrenergic projection to the substantia nigra has a neuroprotective role in the survival of midbrain dopaminergic neurons in facing injuries or perturbations [75]. Thus, the loss of LC-NE inputs can exacerbate the degeneration of SNc DA neurons and the nigrostriatal pathway. Last, LC-NE degeneration may promote the development of α-synuclein aggregation and exacerbate dopaminergic neurodegeneration. Mittal et al. reported that the β2-adrenergic receptor is a regulator of the α-synuclein encoding gene (*SNCA*) and that chronic activation β2 receptors lowers the risk of PD in humans and protects SNc DA neurons in animal models of PD [76]. The degeneration of LC-NE projections to motor regions of the brain plays an important role in developing motor deficits in PD. However, our mechanistic understanding of circuit changes following noradrenergic degeneration remains in its infancy.

## Conclusion

It is clear that LC-NE neurons are affected at early stages of PD and contribute to the manifestation of various nonmotor and motor deficits in people with PD. The degeneration of LC-NE neurons and the associated detrimental impact have been well documented by both clinical and preclinical studies using neuropathological, neuroanatomical, physiological, and behavioral approaches. However, our mechanistic understanding of the detrimental impact of LC-NE degeneration on PD progression and the pathophysiology of Parkinsonian symptoms remains limited. Building our knowledge and lessons from the dopaminergic degeneration and basal ganglia circuits, it is reasonable to expect that future research will provide more information regarding the functional impact of LC-NE degeneration on the cerebral cortex. Cortical dysfunction and its abnormal interaction with subcortical regions can underlie the manifestation of various nonmotor deficits in PD. With the available advanced research technologies, these studies may aim to answer the following questions:

1. How does LC-NE degeneration affect cellular and synaptic function in the cerebral cortex through cell- and input-specific mechanisms? What is the overall impact of LC-NE degeneration on cerebral cortical output at network levels?
2. What are the secondary cortical circuitry compensations for LC-NE degeneration, and how do such compensatory changes play a role in manifesting various PD symptoms?
3. How does LC-NE degeneration affect cortical circuitry function by engaging NE-DA interaction in the cerebral cortex?
4. How do cellular and synaptic level changes associated with LC-NE degeneration lead to mesoscopic level alterations of cortical circuits and the manifestation of motor and nonmotor deficits in PD?
5. Can early intervention of noradrenergic degeneration slow down dopaminergic degeneration or modify the overall progression of PD?

A better understanding of the impact of LC-NE neurodegeneration on cortical circuits is essential to PD pathophysiology research. It can provide new avenues to treat various nonmotor deficits and dopamine-nonresponsive motor symptoms (e.g., freezing of gait) in PD patients.

## References

[1] Schwarz LA, Miyamichi K, Gao XJ, Beier KT, Weissbourd B, DeLoach KE, et al. Viral-genetic tracing of the input–output organization of a central noradrenaline circuit. Nature. 2015;524:88–92. 26131933 10.1038/nature14600 PMC4587569

[2] Swanson LW. The locus coeruleus: a cytoarchitectonic, Golgi and immunohistochemical study in the albino rat. Brain Res. 1976;110:39–56. 776360 10.1016/0006-8993(76)90207-9

[3] Baker KG, Tork I, Hornung JP, Halasz P. The human locus coeruleus complex: an immunohistochemical and three dimensional reconstruction study. Exp Brain Res. 1989;77:257–70. 2571514 10.1007/BF00274983

[4] German DC, Walker BS, Manaye K, Smith WK, Woodward DJ, North AJ. The human locus coeruleus: computer reconstruction of cellular distribution. J Neurosci. 1988;8:1776–88. 3367220 10.1523/JNEUROSCI.08-05-01776.1988 PMC6569207

[5] Perez DM. alpha(1)-Adrenergic receptors in neurotransmission, synaptic plasticity, and cognition. Front Pharmacol. 2020;11:581098. 33117176 10.3389/fphar.2020.581098 PMC7553051

[6] Poe GR, Foote S, Eschenko O, Johansen JP, Bouret S, Aston-Jones G, et al. Locus coeruleus: a new look at the blue spot. Nat Rev Neurosci. 2020;21:644–59. 32943779 10.1038/s41583-020-0360-9 PMC8991985

[7] Berridge CW, Spencer RC. Differential cognitive actions of norepinephrine a2 and a1 receptor signaling in the prefrontal cortex. Brain Res. 2016;1641:189–96. 26592951 10.1016/j.brainres.2015.11.024 PMC4876052

[8] Aston-Jones G, Waterhouse B. Locus coeruleus: from global projection system to adaptive regulation of behavior. Brain Res. 2016;1645:75–8. 26969408 10.1016/j.brainres.2016.03.001 PMC4969192

[9] Ranjbar-Slamloo Y, Fazlali Z. Dopamine and noradrenaline in the brain; overlapping or dissociate functions? Front Mol Neurosci. 2019;12:334. 32038164 10.3389/fnmol.2019.00334 PMC6986277

[10] Weinshenker D. Long road to ruin: noradrenergic dysfunction in neurodegenerative disease. Trends Neurosci. 2018;41:211–23. 29475564 10.1016/j.tins.2018.01.010 PMC5878728

[11] Paredes-Rodriguez E, Vegas-Suarez S, Morera-Herreras T, Deurwaerdere PD, Miguelez C. The noradrenergic system in Parkinson’s disease. Front Pharmacol. 2020;11:435. 32322208 10.3389/fphar.2020.00435 PMC7157437

[12] Ray Chaudhuri K, Leta V, Bannister K, Brooks DJ, Svenningsson P. The noradrenergic subtype of Parkinson disease: from animal models to clinical practice. Nat Rev Neurol. 2023;19:333–45. 37142796 10.1038/s41582-023-00802-5

[13] Sara SJ, Bouret S. Orienting and reorienting: the locus coeruleus mediates cognition through arousal. Neuron. 2012;76:130–41. 23040811 10.1016/j.neuron.2012.09.011

[14] Carter ME, Yizhar O, Chikahisa S, Nguyen H, Adamantidis A, Nishino S, et al. Tuning arousal with optogenetic modulation of locus coeruleus neurons. Nat Neurosci. 2010;13:1526–33. 21037585 10.1038/nn.2682 PMC3174240

[15] Su Z, Cohen JY. Two types of locus coeruleus norepinephrine neurons drive reinforcement learning. Preprint at 10.1101/2022.12.08.519670.

[16] Totah NK, Neves RM, Panzeri S, Logothetis NK, Eschenko O. The locus coeruleus is a complex and differentiated neuromodulatory system. Neuron. 2018;99:1055–68.e6. 30122373 10.1016/j.neuron.2018.07.037

[17] Uematsu A, Tan BZ, Ycu EA, Cuevas JS, Koivumaa J, Junyent F, et al. Modular organization of the brainstem noradrenaline system coordinates opposing learning states. Nat Neurosci. 2017;20:1602–11. 28920933 10.1038/nn.4642

[18] Hirschberg S, Li Y, Randall A, Kremer EJ, Pickering AE. Functional dichotomy in spinal- vs prefrontal-projecting locus coeruleus modules splits descending noradrenergic analgesia from ascending aversion and anxiety in rats. Elife. 2017;6:e29808. 10.7554/eLife.29808 PMC5653237 29027903

[19] Chandler DJ, Gao WJ, Waterhouse BD. Heterogeneous organization of the locus coeruleus projections to prefrontal and motor cortices. Proc Natl Acad Sci USA. 2014;111:6816–21. 24753596 10.1073/pnas.1320827111 PMC4020069

[20] McKinney A, Hu M, Hoskins A, Mohammadyar A, Naeem N, Jing J, et al. Cellular composition and circuit organization of the locus coeruleus of adult mice. Elife. 2023;12:e80100. 10.7554/eLife.80100 PMC9934863 36734517

[21] Seguela P, Watkins KC, Geffard M, Descarries L. Noradrenaline axon terminals in adult rat neocortex: an immunocytochemical analysis in serial thin sections. Neuroscience. 1990;35:249–64. 2116602 10.1016/0306-4522(90)90079-j

[22] Lewis DA, Morrison JH. Noradrenergic innervation of monkey prefrontal cortex: a dopamine-beta-hydroxylase immunohistochemical study. J Comp Neurol. 1989;282:317–30. 2715385 10.1002/cne.902820302

[23] Goldman-Rakic PS, Lidow MS, Gallager DW. Overlap of dopaminergic, adrenergic, and serotoninergic receptors and complementarity of their subtypes in primate prefrontal cortex. J Neurosci. 1990;10:2125–38. 2165520 10.1523/JNEUROSCI.10-07-02125.1990 PMC6570394

[24] Santana N, Artigas F. Laminar and cellular distribution of monoamine receptors in rat medial prefrontal cortex. Front Neuroanat. 2017;11:87. 29033796 10.3389/fnana.2017.00087 PMC5625028

[25] Lee M, Mueller A, Moore T. Differences in noradrenaline receptor expression across different neuronal subtypes in macaque frontal eye field. Front Neuroanat. 2020;14:574130. 33328901 10.3389/fnana.2020.574130 PMC7732642

[26] Dembrow NC, Chitwood RA, Johnston D. Projection-specific neuromodulation of medial prefrontal cortex neurons. J Neurosci. 2010;30:16922–37. 21159963 10.1523/JNEUROSCI.3644-10.2010 PMC3075873

[27] Datta D, Yang ST, Galvin VC, Solder J, Luo F, Morozov YM, et al. Noradrenergic alpha1-adrenoceptor actions in the primate dorsolateral prefrontal cortex. J Neurosci. 2019;39:2722–34. 30755491 10.1523/JNEUROSCI.2472-18.2019 PMC6445993

[28] Mitrano DA, Schroeder JP, Smith Y, Cortright JJ, Bubula N, Vezina P, et al. alpha-1 Adrenergic receptors are localized on presynaptic elements in the nucleus accumbens and regulate mesolimbic dopamine transmission. Neuropsychopharmacology. 2012;37:2161–72. 22588352 10.1038/npp.2012.68 PMC3398716

[29] Santana N, Mengod G, Artigas F. Expression of alpha(1)-adrenergic receptors in rat prefrontal cortex: cellular co-localization with 5-HT_2A_ receptors. Int J Neuropsychopharmacol. 2013;16:1139–51. 23195622 10.1017/S1461145712001083

[30] Kawaguchi Y, Shindou T. Noradrenergic excitation and inhibition of GABAergic cell types in rat frontal cortex. J Neurosci. 1998;18:6963–76. 9712665 10.1523/JNEUROSCI.18-17-06963.1998 PMC6792977

[31] Wang M, Ramos BP, Paspalas CD, Shu Y, Simen A, Duque A, et al. Alpha2A-adrenoceptors strengthen working memory networks by inhibiting cAMP-HCN channel signaling in prefrontal cortex. Cell. 2007;129:397–410. 17448997 10.1016/j.cell.2007.03.015

[32] Joyce MKP, Yang S, Morin K, Duque A, Arellano J, Datta D, et al. beta1-adrenoceptor expression on GABAergic interneurons in primate dorsolateral prefrontal cortex: potential role in stress-induced cognitive dysfunction. Neurobiol Stress. 2024;30:100628. 38550854 10.1016/j.ynstr.2024.100628 PMC10973161

[33] Liu Y, Liang X, Ren WW, Li BM. Expression of beta1- and beta2-adrenoceptors in different subtypes of interneurons in the medial prefrontal cortex of mice. Neuroscience. 2014;257:149–57. 24215978 10.1016/j.neuroscience.2013.10.078

[34] Devoto P, Flore G, Pira L, Longu G, Gessa GL. Alpha2-adrenoceptor mediated co-release of dopamine and noradrenaline from noradrenergic neurons in the cerebral cortex. J Neurochem. 2004;88:1003–9. 14756822 10.1046/j.1471-4159.2003.02239.x

[35] Devoto P, Flore G, Longu G, Pira L, Gessa GL. Origin of extracellular dopamine from dopamine and noradrenaline neurons in the medial prefrontal and occipital cortex. Synapse. 2003;50:200–5. 14515337 10.1002/syn.10264

[36] Devoto P, Flore G, Pani L, Gessa GL. Evidence for co-release of noradrenaline and dopamine from noradrenergic neurons in the cerebral cortex. Mol Psychiatry. 2001;6:657–64. 11673793 10.1038/sj.mp.4000904

[37] Sesack SR, Hawrylak VA, Matus C, Guido MA, Levey AI. Dopamine axon varicosities in the prelimbic division of the rat prefrontal cortex exhibit sparse immunoreactivity for the dopamine transporter. J Neurosci. 1998;18:2697–708. 9502827 10.1523/JNEUROSCI.18-07-02697.1998 PMC6793120

[38] Sanchez-Soto M, Casado-Anguera V, Yano H, Bender BJ, Cai NS, Moreno E, et al. alpha(2A)- and alpha(2C)-Adrenoceptors as potential targets for dopamine and dopamine receptor ligands. Mol Neurobiol. 2018;55:8438–54. 29552726 10.1007/s12035-018-1004-1 PMC6143434

[39] Kobayashi K, Shikano K, Kuroiwa M, Horikawa M, Ito W, Nishi A, et al. Noradrenaline activation of hippocampal dopamine D(1) receptors promotes antidepressant effects. Proc Natl Acad Sci USA. 2022;119:e2117903119. 35939697 10.1073/pnas.2117903119 PMC9388128

[40] Moron JA, Brockington A, Wise RA, Rocha BA, Hope BT. Dopamine uptake through the norepinephrine transporter in brain regions with low levels of the dopamine transporter: evidence from knock-out mouse lines. J Neurosci. 2002;22:389–95. 11784783 10.1523/JNEUROSCI.22-02-00389.2002 PMC6758674

[41] Mitrano DA, Pare JF, Smith Y, Weinshenker D. D1-dopamine and alpha1-adrenergic receptors co-localize in dendrites of the rat prefrontal cortex. Neuroscience. 2014;258:90–100. 24231738 10.1016/j.neuroscience.2013.11.002 PMC3913162

[42] Goldman-Rakic PS. Cellular basis of working memory. Neuron. 1995;14:477–85. 7695894 10.1016/0896-6273(95)90304-6

[43] Fuster JM, Alexander GE. Neuron activity related to short-term memory. Science. 1971;173:652–4. 4998337 10.1126/science.173.3997.652

[44] Kubota K, Niki H. Prefrontal cortical unit activity and delayed alternation performance in monkeys. J Neurophysiol. 1971;34:337–47. 4997822 10.1152/jn.1971.34.3.337

[45] Zhang Z, Cordeiro Matos S, Jego S, Adamantidis A, Seguela P. Norepinephrine drives persistent activity in prefrontal cortex via synergistic alpha1 and alpha2 adrenoceptors. PLoS One. 2013;8:e66122. 23785477 10.1371/journal.pone.0066122 PMC3681776

[46] Luo F, Zheng J, Sun X, Tang H. Inward rectifier K^+^ channel and T-type Ca^2+^ channel contribute to enhancement of GABAergic transmission induced by beta(1)-adrenoceptor in the prefrontal cortex. Exp Neurol. 2017;288:51–61. 27840071 10.1016/j.expneurol.2016.11.007

[47] Arnsten AF. Stress signalling pathways that impair prefrontal cortex structure and function. Nat Rev Neurosci. 2009;10:410–22. 19455173 10.1038/nrn2648 PMC2907136

[48] Waterhouse BD, Predale HK, Plummer NW, Jensen P, Chandler DJ. Probing the structure and function of locus coeruleus projections to CNS motor centers. Front Neural Circuits. 2022;16:895481. 36247730 10.3389/fncir.2022.895481 PMC9556855

[49] Plummer NW, Chandler DJ, Powell JM, Scappini EL, Waterhouse BD, Jensen P. An intersectional viral-genetic method for fluorescent tracing of axon collaterals reveals details of noradrenergic locus coeruleus structure. Eneuro. 2020;7:ENEURO.0010-20.2020. 10.1523/ENEURO.0010-20.2020 PMC7294462 32354756

[50] Sheets PL, Suter BA, Kiritani T, Chan CS, Surmeier DJ, Shepherd GM, et al. expression in mouse motor cortex: I(h)-dependent synaptic integration as a candidate microcircuit mechanism involved in motor control. J Neurophysiol. 2011;106:2216–31. 21795621 10.1152/jn.00232.2011 PMC3214092

[51] Schiemann J, Puggioni P, Dacre J, Pelko M, Domanski A, van Rossum Mark CW, et al. Cellular mechanisms underlying behavioral state-dependent bidirectional modulation of motor cortex output. Cell Rep. 2015;11:1319–30. 25981037 10.1016/j.celrep.2015.04.042 PMC4451462

[52] Tseng CT, Welch HF, Gi AL, Kang EM, Mamidi T, Pydimarri S, et al. Frequency specific optogenetic stimulation of the locus coeruleus induces task-relevant plasticity in the motor cortex. J Neurosci. 2024;44e1528232023. 10.1523/JNEUROSCI.1528-23.2023 PMC10869157 38124020

[53] Suter BA, Migliore M, Shepherd GMG. Intrinsic electrophysiology of mouse corticospinal neurons: a class-specific triad of spike-related properties. Cereb Cortex. 2013;23:1965–77. 22761308 10.1093/cercor/bhs184 PMC3698370

[54] Zarow C, Lyness SA, Mortimer JA, Chui HC. Neuronal loss is greater in the locus coeruleus than nucleus basalis and substantia nigra in Alzheimer and Parkinson diseases. Arch Neurol. 2003;60:337–41. 12633144 10.1001/archneur.60.3.337

[55] Vermeiren Y, De Deyn PP. Targeting the norepinephrinergic system in Parkinson’s disease and related disorders: the locus coeruleus story. Neurochem Int. 2017;102:22–32. 27899296 10.1016/j.neuint.2016.11.009

[56] Braak H, Del Tredici K, Rüb U, de Vos RA, Jansen Steur EN, Braak E. Staging of brain pathology related to sporadic Parkinson’s disease. Neurobiol Aging. 2003;24:197–211. 12498954 10.1016/s0197-4580(02)00065-9

[57] McMillan PJ, White SS, Franklin A, Greenup JL, Leverenz JB, Raskind MA, et al. Differential response of the central noradrenergic nervous system to the loss of locus coeruleus neurons in Parkinson’s disease and Alzheimer’s disease. Brain Res. 2011;1373:240–52. 21147074 10.1016/j.brainres.2010.12.015 PMC3038670

[58] Bertrand E, Lechowicz W, Szpak GM, Dymecki J. Qualitative and quantitative analysis of locus coeruleus neurons in Parkinson’s disease. Folia Neuropathol. 1997;35:80–6. 9377080

[59] German DC, Manaye KF, White CL 3rd, Woodward DJ, McIntire DD, Smith WK, et al. Disease-specific patterns of locus coeruleus cell loss. Ann Neurol. 1992;32:667–76. 1449247 10.1002/ana.410320510

[60] Henrich MT, Geibl FF, Lee B, Chiu WH, Koprich JB, Brotchie JM, et al. A53T-alpha-synuclein overexpression in murine locus coeruleus induces Parkinson’s disease-like pathology in neurons and glia. Acta Neuropathol Commun. 2018;6:39. 29747690 10.1186/s40478-018-0541-1 PMC5946574

[61] Butkovich LM, Houser MC, Chalermpalanupap T, Porter-Stransky KA, Iannitelli AF, Boles JS, et al. Transgenic mice expressing human α-synuclein in noradrenergic neurons develop locus ceruleus pathology and nonmotor features of Parkinson’s disease. J Neurosci. 2020;40:7559–76. 32868457 10.1523/JNEUROSCI.1468-19.2020 PMC7511194

[62] Szot P, Franklin A, Miguelez C, Wang Y, Vidaurrazaga I, Ugedo L, et al. Depressive-like behavior observed with a minimal loss of locus coeruleus (LC) neurons following administration of 6-hydroxydopamine is associated with electrophysiological changes and reversed with precursors of norepinephrine. Neuropharmacology. 2016;101:76–86. 26362360 10.1016/j.neuropharm.2015.09.003 PMC4782767

[63] Iannitelli AF, Kelberman MA, Lustberg DJ, Korukonda A, McCann KE, Mulvey B, et al. The neurotoxin DSP-4 dysregulates the locus coeruleus-norepinephrine system and recapitulates molecular and behavioral aspects of prodromal neurodegenerative disease. Eneuro. 2023;10:ENEURO.0483-22.2022. 10.1523/ENEURO.0483-22.2022 PMC9829100 36635251

[64] Cullen KP, Grant LM, Kelm-Nelson CA, Brauer AFL, Bickelhaupt LB, Russell JA, et al. Pink1 ^-/-^ rats show early-onset swallowing deficits and correlative brainstem pathology. Dysphagia. 2018;33:749–58. 29713896 10.1007/s00455-018-9896-5 PMC6207473

[65] Giaime E, Tong Y, Wagner LK, Yuan Y, Huang G, Shen J. Age-dependent dopaminergic neurodegeneration and impairment of the autophagy-lysosomal pathway in LRRK-deficient mice. Neuron. 2017;96:796–807.e6. 29056298 10.1016/j.neuron.2017.09.036 PMC5693787

[66] Scatton B, Javoy-Agid F, Rouquier L, Dubois B, Agid Y. Reduction of cortical dopamine, noradrenaline, serotonin and their metabolites in Parkinson’s disease. Brain Res. 1983;275:321–8. 6626985 10.1016/0006-8993(83)90993-9

[67] Xing B, Li YC, Gao WJ. Norepinephrine versus dopamine and their interaction in modulating synaptic function in the prefrontal cortex. Brain Res. 2016;1641:217–33. 26790349 10.1016/j.brainres.2016.01.005 PMC4879059

[68] Cash R, Ruberg M, Raisman R, Agid Y. Adrenergic receptors in Parkinson’s disease. Brain Res. 1984;322:269–75. 6095967 10.1016/0006-8993(84)90117-3

[69] Matschke LA, Komadowski MA, Stohr A, Lee B, Henrich MT, Griesbach M, et al. Enhanced firing of locus coeruleus neurons and SK channel dysfunction are conserved in distinct models of prodromal Parkinson’s disease. Sci Rep. 2022;12:3180. 35210472 10.1038/s41598-022-06832-1 PMC8873463

[70] Harro J, Pahkla R, Modiri AR, Harro M, Kask A, Oreland L. Dose-dependent effects of noradrenergic denervation by DSP-4 treatment on forced swimming and beta-adrenoceptor binding in the rat. J Neural Transm. 1999;106:619–29. 10907722 10.1007/s007020050184

[71] Johnson RD, Iuvone PM, Minneman KP. Regulation of alpha-1 adrenergic receptor density and functional responsiveness in rat brain. J Pharmacol Exp Ther. 1987;242:842–9. 2821227

[72] Rommelfanger KS, Edwards GL, Freeman KG, Liles LC, Miller GW, Weinshenker D. Norepinephrine loss produces more profound motor deficits than MPTP treatment in mice. Proc Natl Acad Sci USA. 2007;104:13804–9. 17702867 10.1073/pnas.0702753104 PMC1959463

[73] Guo L, Xiong H, Kim JI, Wu YW, Lalchandani RR, Cui Y, et al. Dynamic rewiring of neural circuits in the motor cortex in mouse models of Parkinson’s disease. Nat Neurosci. 2015;18:1299–309. 26237365 10.1038/nn.4082 PMC4551606

[74] Chu H-Y, Smith Y, Lytton WW, Grafton S, Villalba R, Masilamoni G, et al. Dysfunction of motor cortices in Parkinson’s disease. Cereb Cortex. 2024;34:bhae294. 10.1093/cercor/bhae294 PMC11281850 39066504

[75] Jovanovic P, Wang Y, Vit JP, Novinbakht E, Morones N, Hogg E, et al. Sustained chemogenetic activation of locus coeruleus norepinephrine neurons promotes dopaminergic neuron survival in synucleinopathy. PLoS One. 2022;17:e0263074. 35316276 10.1371/journal.pone.0263074 PMC8939823

[76] Mittal S, Bjornevik K, Im DS, Flierl A, Dong X, Locascio JJ, et al. beta2-Adrenoreceptor is a regulator of the alpha-synuclein gene driving risk of Parkinson’s disease. Science. 2017;357:891–8. 28860381 10.1126/science.aaf3934 PMC5761666

